# Cardiac strength-interval curves calculated using a bidomain tissue with a parsimonious ionic current

**DOI:** 10.1371/journal.pone.0171144

**Published:** 2017-02-21

**Authors:** Suran K. Galappaththige, Richard A. Gray, Bradley J. Roth

**Affiliations:** 1 Department of Physics, Oakland University, Rochester, Michigan, United States of America; 2 Center for Devices and Radiological Health, Food and Drug Administration, Silver Spring, Maryland, United States of America; Universiteit Gent, BELGIUM

## Abstract

The strength-interval curve plays a major role in understanding how cardiac tissue responds to an electrical stimulus. This complex behavior has been studied previously using the bidomain formulation incorporating the Beeler-Reuter and Luo-Rudy dynamic ionic current models. The complexity of these models renders the interpretation and extrapolation of simulation results problematic. Here we utilize a recently developed parsimonious ionic current model with only two currents—a sodium current that activates rapidly upon depolarization *I*_*Na*_ and a time-independent inwardly rectifying repolarization current *I*_*K*_—which reproduces many experimentally measured action potential waveforms. Bidomain tissue simulations with this ionic current model reproduce the distinctive dip in the anodal (but not cathodal) strength-interval curve. Studying model variants elucidates the necessary and sufficient physiological conditions to predict the polarity dependent dip: a voltage and time dependent *I*_*Na*_, a nonlinear rectifying repolarization current, and bidomain tissue with unequal anisotropy ratios.

## Introduction

An in-depth understanding of cardiac excitability is critical to the development of pacemakers and defibrillators. Modeling the dynamics of wave excitation in cardiac tissue is a way to predict the complex behavior of the tissue in response to an applied stimulus. Many models have been used to predict this behavior; typically these models have numerous parameters and ion currents, and each model has its own strengths and weaknesses [1]. The complexity of these models make it difficult to identify the relative role of parameters and variables on cell behavior such as the action potential duration (APD) and the excitation threshold. Other “phenomenological” models are simpler with fewer parameters, but are less realistic, and do not allow a link to detailed physiology such as voltage clamp experiments.

The strength-interval (SI) curve is a useful way to characterize the behavior of cardiac tissue in response to a stimulus [2]. Two stimuli are applied separated by an interval of time. The first (S_1_) triggers an action potential, and the second (S_2_) probes the tissue excitability as the tissue recovers from S_1_ refractoriness. Previous experiments [3], as well as simulations using the bidomain model [4] with the Beeler-Reuter model [5], have shown the mechanisms of excitation underlying cathodal and anodal SI curves are cathode make, cathode break, anode make, and anode break. “Cathode” and “anode” refer to the polarity of the stimulus electrode, and “make” and “break” to the mechanism of tissue excitation; make is when excitation begins directly from depolarized tissue following the *onset* of the stimulus pulse, and break is when it begins (usually indirectly through hyperpolarized tissue) *following* the termination of the pulse.

Similar modeling studies [6], including one using the Luo-Rudy dynamic model [7, 8], analyzed the “dip” in the anodal SI curve. Typically, the SI curve has negative slope, indicating that the S_2_ threshold is lower at longer intervals when the tissue has recovered from S_1_ refractoriness. For anodal stimulation, however, part of the SI curve has a positive slope, indicating that S_2_ excitation is easier when the tissue is less recovered. This counterintuitive behavior is prominent in the break section of the anodal SI curve, and is suppressed or absent from the cathodal SI curve. One proposed mechanism responsible for the dip is that following the S_2_ stimulus, the tissue surrounding the anode is hyperpolarized and excitable. The limiting factor determining the threshold is how well this excitable tissue interacts with nearby depolarization that can be produced by either the S_2_ stimulus or the previous S_1_ action potential. As the interval is shortened, more S_1_ depolarization is present so less S_2_ depolarization is required, lowering the threshold S_2_ stimulus strength. Thus, the dip arises from the electrotonic interaction of excitable tissue with surrounding depolarization [2,6,7]. Other studies have claimed that the mechanism of the dip is caused by calcium dynamics [9] or is an intrinsic property of the membrane current [10,11].

A recently developed parsimonious ionic model [12, 13] reproduces many experimentally measured characteristics of ventricular action potentials using only three variables, two ion currents, and eleven parameters. The advantage of a parsimonious model is that it highlights essential features of the membrane kinetics while not getting bogged down in nonessential details. The purpose of this manuscript is to combine the parsimonious ionic model with the bidomain formulation to study the events responsible for the shape of the strength interval curve. Specifically, we explore which features of the parsimonious model are essential for predicting the anomalous dip in the anodal SI curve. Our hypothesis is that a parsimonious model coupled with the bidomain formulation is sufficient to predict the shapes of the anodal and cathodal SI curves, and that the nonlinearity of the repolarization current is necessary for the dip in the anodal SI curve.

## Results

### Space-Clamped model

Anodal stimulation (hyperpolarization) did not cause excitation in the space-clamped parsimonious model. Space-clamped cathodal stimulation (depolarization) resulted in supernormal excitation immediately following a previous S_1_ action potential, which may arise because the transmembrane potential has not yet returned to rest and therefore less depolarization is required to reach threshold, even though *I*_*Na*_ is not fully inactivated [14, 15].

### Parsimonious ionic current model with bidomain tissue model

**Fig 1:**
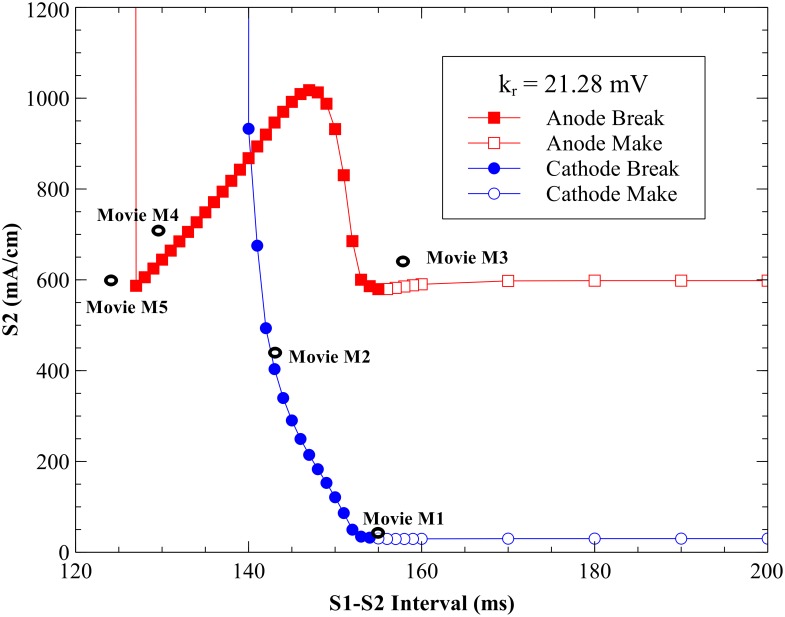
The cathodal and anodal strength-interval curves for the parsimonious ionic model. Cathodal (blue) and anodal (red), with *k_r_* = 21.28 mV, *g_K_* = 0.3 mS cm^-2^, and *E_K_* = -83 mV. The simulations included as movies in the supplementary information are indicated by black open ovals.

The strength interval curves for both cathodal and anodal stimuli applied through a unipolar electrode for a 2 ms pulse are shown in Fig 1, using the standard model parameters [12, 13]. The S_1_-S_2_ interval is defined as the time between the start of the S_1_ stimulus pulse (at time 0) and the start of the S_2_ pulse. These tissue simulations using the bidomain model with unequal anisotropy ratios predict a complicated transmembrane potential distribution with adjacent regions of depolarization and hyperpolarization [3,16] for both anodal and cathodal stimulation.

#### Cathodal SI curve

**Fig 2:**
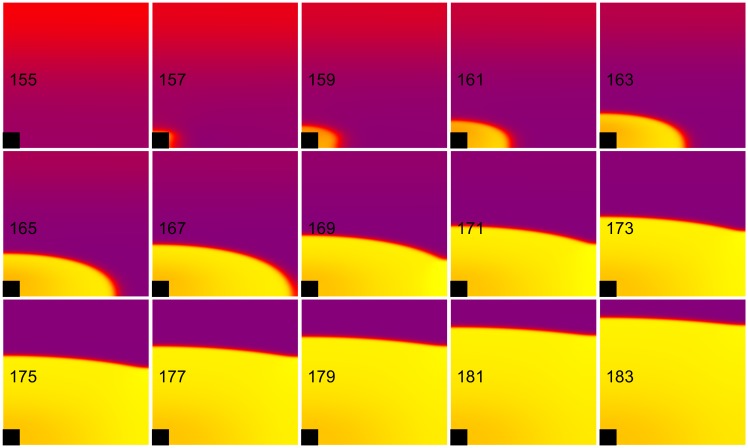
Cathode make excitation. The transmembrane potential is shown as a function of *x* (horizontal, parallel to the fibers) and *y* (vertical, perpendicular to the fibers). The black rectangle is the electrode and the number in each frame is the time in ms. S_1_-S_2_ interval = 155 ms, S_2_ stimulus = 33 mA/cm. These frames are taken from the S1 Movie (see supplementary files). The color scale for this and other plots of transmembrane potential is shown in the movie; purple is rest, yellow is depolarized, and blue is hyperpolarized.

**Fig 3:**
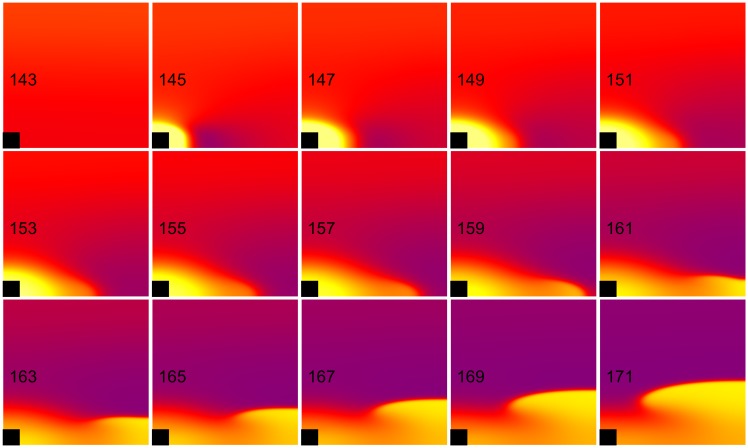
Cathode break excitation. The transmembrane potential is shown as a function of *x* (horizontal, parallel to the fibers) and *y* (vertical, perpendicular to the fibers). The black rectangle is the electrode and the number in each frame is the time in ms. S_1_-S_2_ interval = 143 ms, S_2_ stimulus = 435 mA/cm. These frames are taken from the S2 Movie (see supplementary files).

The SI curve for cathodal stimulation (Fig 1, blue curve) shows a decrease in threshold S_2_ stimulus strength as the tissue is recovering from the S_1_ action potential. At long intervals (rheobase), the cathodal threshold is 30 mA/cm. At an interval of 155 ms the tissue is nearly recovered from S_1_ refractoriness and cathode make excitation originates from the depolarization surrounding the cathode. Details of this behavior are shown in Fig 2 and S1 Movie (interval = 155 ms, S_2_ stimulus = 33 mA/cm; see supplementary information). At shorter intervals cathode break excitation occurs. The tissue is still refractory at 143 ms when the S_2_ pulse begins. During and immediately after the S_2_ stimulus the depolarization is extensive and hyperpolarization along the fiber direction is evident (the “virtual anode”). Break excitation occurs following the end of the S_2_ pulse, when the depolarization surrounding the cathode diffuses into the hyperpolarized virtual anode, exciting a wave front that initially propagates through the hyperpolarized and therefore excitable tissue along the fiber axis (Fig 3 and S2 Movie, 143 ms, 435 mA/cm). At less than 140 ms, the tissue is unexcitable for any S_2_ strength (absolute refractory).

#### Anodal SI curve

The anodal SI curve (Fig 1, red curve) has a prominent dip (a local minimum created by an abrupt drop at 127 ms followed by a region of positive slope), as seen in other models [2,6,7]. The threshold value of the anodal stimulus at the minimum of the dip is 587 mA/cm. For S_1_-S_2_ intervals below 127 ms the tissue is unexcitable. The maximum threshold value is 1017 mA/cm recorded at 147 ms. The range from 155 to 160 ms contains a supernormal period similar to that in the space-clamped simulations, during which the threshold dips to at most 3% below its asymptotic long-interval (rheobase) value of 598 mA/cm.

**Fig 4:**
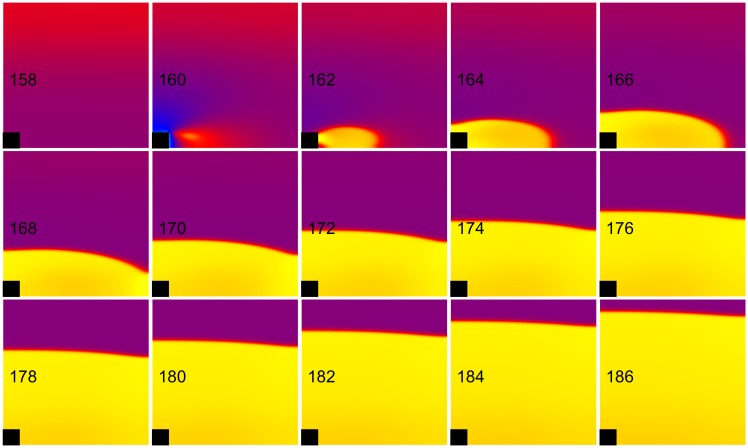
Anode make excitation. The transmembrane potential is shown as a function of *x* (horizontal, parallel to the fibers) and *y* (vertical, perpendicular to the fibers). The black rectangle is the electrode and the number in each frame is the time in ms. S_1_-S_2_ interval = 158 ms, S_2_ stimulus = 640 mA/cm. These frames are taken from the S3 Movie (see supplementary files).

**Fig 5:**
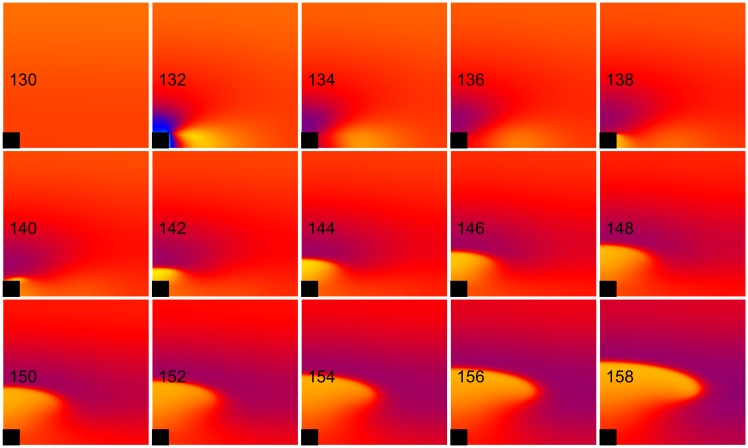
Anode break excitation. The transmembrane potential is shown as a function of *x* (horizontal, parallel to the fibers) and *y* (vertical, perpendicular to the fibers). The black rectangle is the electrode and the number in each frame is the time in ms. S_1_-S_2_ interval = 130 ms, S_2_ stimulus = 710 mA/cm. These frames are taken from the S4 Movie (see supplementary files).

**Fig 6:**
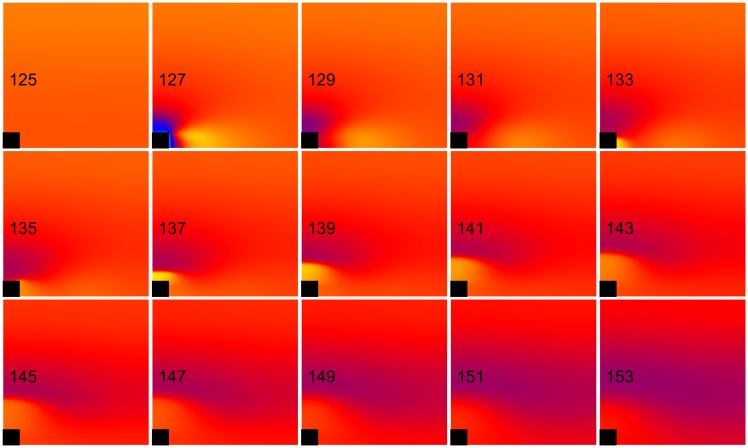
Failed anode break excitation. The transmembrane potential is shown as a function of *x* (horizontal, parallel to the fibers) and *y* (vertical, perpendicular to the fibers). The black rectangle is the electrode and the number in each frame is the time in ms. S_1_-S_2_ interval = 125 ms, S_2_ stimulus = 600 mA/cm. These frames are taken from S5 Movie (see supplementary files).

Anode make excitation occurs at long intervals. The hyperpolarization surrounding the anode plays little role in excitation, and the wave front originates from the depolarized region (the “virtual cathode”) along the fiber direction (Fig 4 and S3 Movie, 158 ms, 640 mA/cm). The supernormal period (155–160 ms) was restricted to the make section of the SI curve. Anode break excitation occurs at shorter intervals. The depolarization along the fiber axis diffuses into the hyperpolarized region surrounding the anode, exciting a wave front that propagates initially perpendicular to the fiber direction (Fig 5 and S4 Movie, 130 ms, 710 mA/cm). At intervals shorter than 127 ms, anode break initiates an action potential but it can only propagate locally near the electrode because the surrounding tissue is too refractory and therefore is incapable of generating a wave that propagates through the heart (Fig 6 and S5 Movie, 125 ms, 600 mA/cm).

### Equal anisotropy ratios

The SI curves were recalculated using equal anisotropy ratios, so no virtual anode was produced during cathodal stimulation and no virtual cathode occurred during anodal stimulation. Anodal stimulation did not excite the tissue. Cathodal stimulation was always by the make mechanism and not the break mechanism.

### Effect of *I*_*K*_ current on strength-interval curves

The repolarization current *I*_*K*_ contains three parameters (see Methods section), each of which modifies the *I*_*K*_ current-voltage curve in a unique way. The conductance *g*_*K*_ scales the magnitude of the *I*_*K*_ current, *E*_*K*_ is the reversal potential, and *k*_*r*_ controls the nonlinearity of the current-voltage curve.

**Fig 7:**
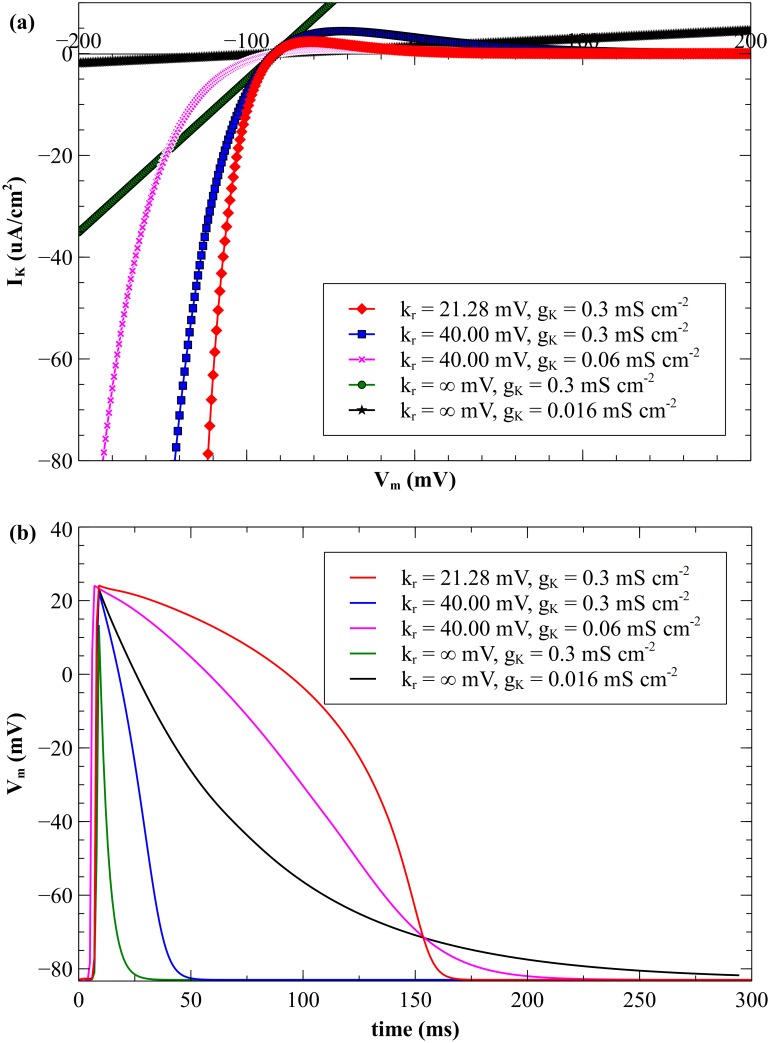
Repolarization current and transmembrane potential for different *k_r_* values. (a) *I_K_* vs *V_m_* and (b) *V_m_* vs time at *x* = 0.35 cm and *y* = 0.1 cm, for different *k_r_* values. The parameters producing the red, purple, and black curves were chosen so the action potential duration is approximately the same.

The variation of *I*_*K*_ with transmembrane potential for different *k*_*r*_ values is shown in Fig 7a. The values for *k*_*r*_ are chosen to show the effect of the *I*_*K*_ current when it is linear (*k*_*r*_ = ∞) and nonlinear (*k*_*r*_ = 21.28 and 40 mV). The value *k*_*r*_ = 21.28 mV corresponds to that used in Gray’s original model [12, 13]. When *g*_*K*_ and *E*_*K*_ are fixed (0.3 mS cm^-2^ and -83 mV), the current-voltage curves have the same intercept and slope for *I*_*K*_ = 0.

#### Cathodal SI curves

**Fig 8:**
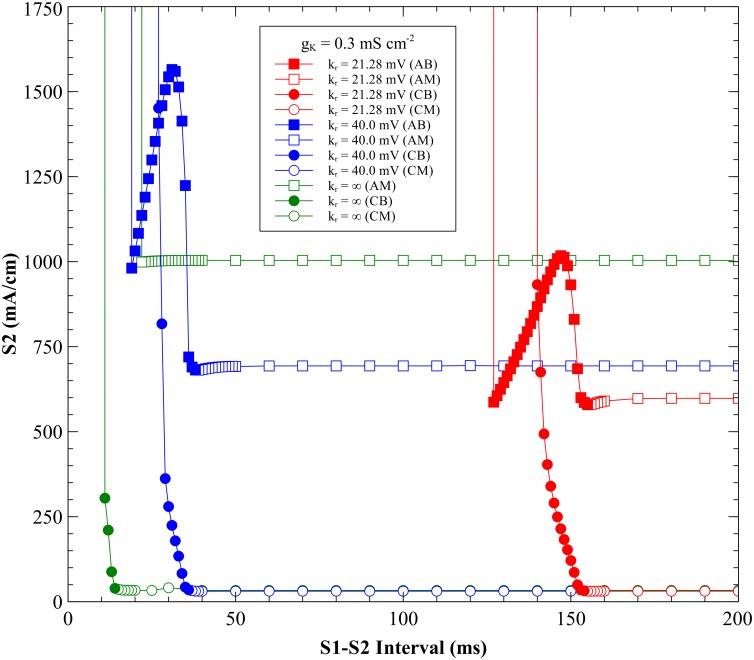
Comparison of SI curves for different *k_r_* values. *k_r_* = 21.28 mV red, *k_r_* = 40 mV blue, and *k_r_* = ∞ green. CM = cathode make (open circles), CB = cathode break (filled circles), AM = anode make (open squares), and AB = anode break (filled squares).

The calculated SI curves are shown in Fig 8. All cathodal SI curves show a decrease in the S_2_ threshold strength as the S_1_-S_2_ interval increases [2]. The cathodal rheobase varies little with *k*_*r*_; for *k*_*r*_ = 21.28 mV the S_2_ threshold is 33 mA/cm, for *k*_*r*_ = 40 mV S_2_ is 30 mA/cm, and for *k*_*r*_ = ∞ S_2_ is 31 mA/cm. The cathodal SI curves show the transition from break (solid symbols) to make (open symbols); for *k*_*r*_ = ∞ the transition occurs at 15 ms, for *k*_*r*_ = 40 mV it occurs at 37 ms, and for *k*_*r*_ = 21.28 mV it occurs at 155 ms. The shift of the SI curve to shorter intervals as *k*_*r*_ increases reflects the time taken by the tissue to recover from refractoriness, where a change in *k*_*r*_ changes the duration of the propagating action potential (Fig 7b). For the cathodal SI curve with *k*_*r*_ = 21.28 mV the tissue is unexcitable below 140 ms, for *k*_*r*_ = 40 mV below 27 ms, and for *k*_*r*_ = ∞ below 11 ms.

#### Anodal SI curves

The *I*_*K*_ current has more of an effect on the shape of the anodal SI curve than on the cathodal curve (Fig 8). For a linear *I*_*K*_ current (*k*_*r*_ = ∞) there is no anode break excitation and no dip in the anodal SI curve. The threshold strength for S_2_ remains almost constant with an increase in S_1_-S_2_ interval and the rheobase strength is highest of all *k*_*r*_ values examined.

The APD and the amplitude of the action potential depend upon *k*_*r*_. With a nonlinear *I*_*K*_ current the anodal SI curve exhibits a transition from break to make excitation; for *k*_*r*_ = 21.28 mV the transition occurs at 155 ms, and for *k*_*r*_ = 40 mV at 38 ms. The longer APD (*k*_*r*_ = 21.28 mV) results in a SI curve with a smaller threshold dip than the short APD (*k*_*r*_ = 40 mV). When *k*_*r*_ = 40 mV, the anodal SI curve has a dip at 19 ms with a stimulus strength of 981 mA/cm. The maximum threshold current is 1565 mA/cm at 31 ms. For *k*_*r*_ = 21.28 mV, anodal SI curve dip is at 127 ms with a strength of 587 mA/cm. Rheobase is highest for *k*_*r*_ = ∞.

### Effect of *k*_*r*_ and *g*_*K*_ parameters on strength-interval curves

**Fig 9:**
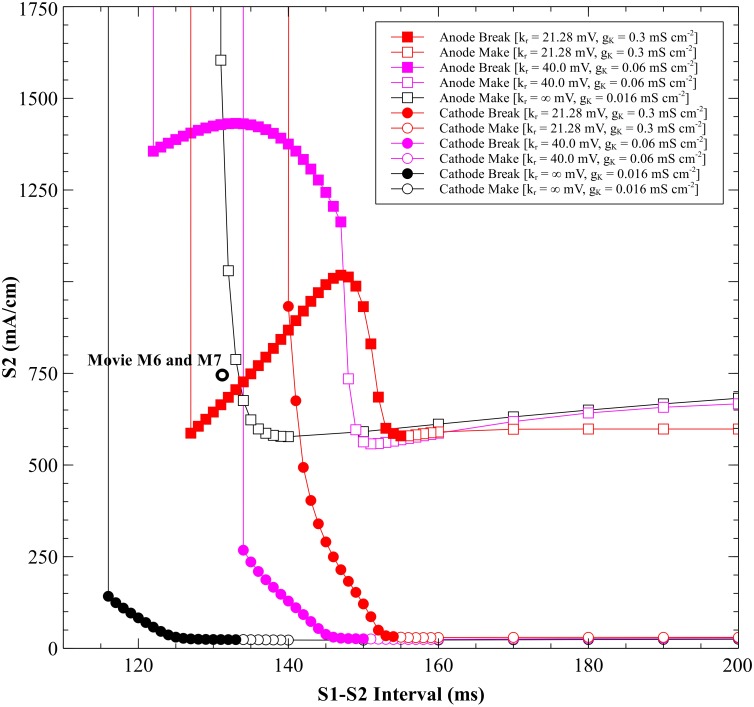
SI curves for different nonlinearities but similar action potential durations. *k_r_* = 21.28 mV and *g_K_* = 0.3 mS cm^-2^ red, *k_r_* = 40 mV and *g_K_* = 0.06 mS cm^-2^ purple, and *k_r_* = ∞ and *g_K_* = 0.016 mS cm^-2^ black. The parameters were chosen so the action potential duration is approximately the same in each case. CM = cathode make (open circles), CB = cathode break (filled circles), AM = anode make (open squares), and AB = anode break (filled squares).

One limitation of varying *k*_*r*_ by itself is that the action potential duration changes dramatically. In order to look at changes in the nonlinearity of *I*_*K*_ without confounding changes in APD, we varied both *k*_*r*_ and *g*_*K*_ simultaneously to keep the APD approximately constant. Fig 7b shows that *k*_*r*_ = 40 mV and *g*_*K*_ = 0.06 mS cm^-2^ produces a triangular shaped action potential, and that *k*_*r*_ = ∞ mV and *g*_*K*_ = 0.016 mS cm^-2^ produces a decaying exponential action potential, both with a duration similar to the standard action potential (*k*_*r*_ = 21.28 mV and *g*_*K*_ = 0.3 mS cm^-2^). Fig 7a shows the variation of the *I*_*K*_ current with membrane potential. The SI curves of these three cases are shown in Fig 9. The anodal strength-interval curve for the linear case (*k*_*r*_ = ∞) does not contain a section for anode break excitation, and does not contain a dip.

**Fig 10:**
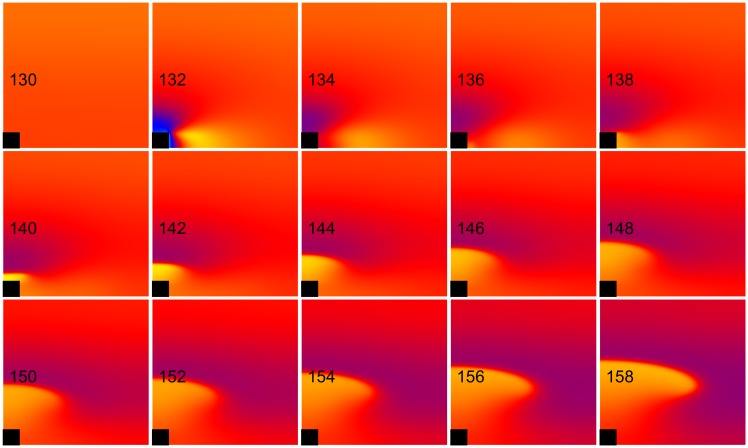
Anode break excitation for the nonlinear case. *k*_*r*_ = 21.28 mV and *g*_*K*_ = 0.3 mS cm^-2^ (normal, nonlinear). The transmembrane potential is shown as a function of *x* (horizontal, parallel to the fibers) and *y* (vertical, perpendicular to the fibers). The black rectangle is the electrode and the number in each frame is the time in ms. S_1_-S_2_ interval = 130 ms, S_2_ stimulus = 750 mA/cm. These frames are taken from S6 Movie (see supplementary files).

**Fig 11:**
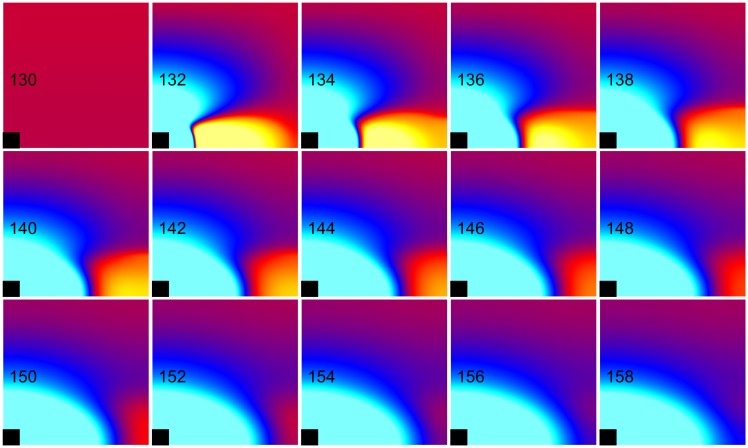
Failed anodal excitation for the linear case. *k*_*r*_ = ∞ and *g*_*K*_ = 0.016 mS cm^-2^ (linear). The transmembrane potential is shown as a function of *x* (horizontal, parallel to the fibers) and *y* (vertical, perpendicular to the fibers). The black rectangle is the electrode and the number in each frame is the time in ms. S_1_-S_2_ interval = 130 ms, S_2_ stimulus = 750 mA/cm. These frames are taken from S7 Movie (see supplementary files).

**Fig 12:**
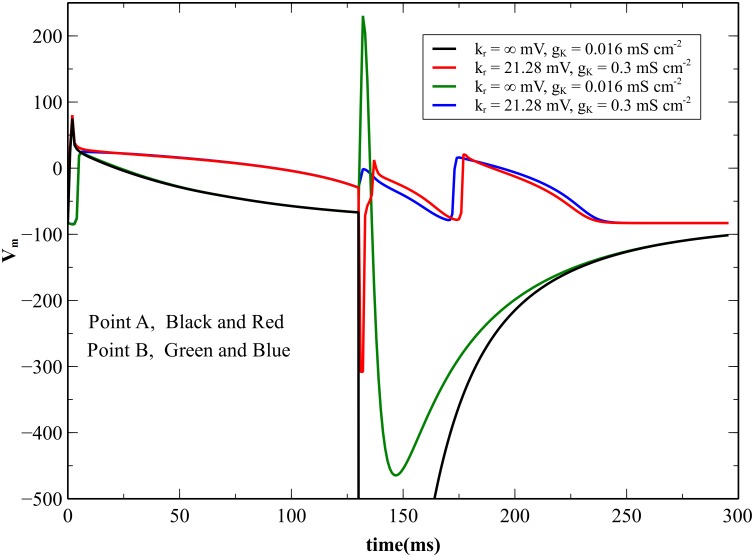
The transmembrane potential as a function of time for nonlinear and linear cases. The transmembrane potential at point A near the anode (*x* = 0.055 cm, *y* = 0.026 cm) and at point B in the virtual cathode (*x* = 0.255 cm, *y* = 0.026 cm), for a normal nonlinear (*k*_*r*_ = 21.28 mV and *g*_*K*_ = 0.3 mS cm^-2^) and a linear (*k*_*r*_ = ∞ mV and *g*_*K*_ = 0.016 mS cm^-2^) repolarization current. The S_2_ stimulus begins at 130 ms.

A nonlinear *I*_*K*_ current is necessary for anode break excitation. To examine why, we performed simulations of anodal excitation for an interval of 130 ms, for *k*_*r*_ = 21.28 mV and g_K_ = 0.3 mS cm^-2^ (normal, nonlinear) and for *k*_*r*_ = ∞ and g_K_ = 0.016 mS cm^-2^ (linear). Fig 10 and S6 Movie show the normal case, and Fig 11 and S7 Movie show the linear case (both 130 ms, 750 mA/cm). Fig 12 shows the time course of the transmembrane potential at two locations, immediately adjacent to the anode (Fig 12a) and at the virtual cathode (Fig 12b). In the normal case, the hyperpolarization surrounding the anode is relatively weak and decays quickly after the stimulus pulse ends, creating an excitable path for the break wave front [5, 6, 17]. In the linear case, the hyperpolarization surrounding the anode is strong and decays slowly. The depolarization does not diffuse into hyperpolarized and excitable tissue. Rather, hyperpolarization diffuses into depolarized and refractory tissue, which does not result in excitation [18]. The nonlinearity of the repolarization current is critical to reducing the extent of the hyperpolarization.

### Effect of *E*_*K*_ on strength-interval curve

**Fig 13:**
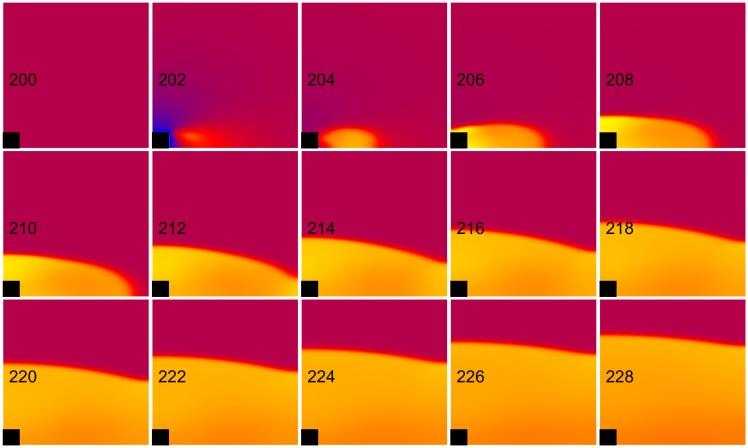
Anode make/break excitation for an elevated extracellular potassium ion concentration. *E*_*K*_ = -69 mV. The transmembrane potential is shown as a function of *x* (horizontal, parallel to the fibers) and *y* (vertical, perpendicular to the fibers). The black rectangle is the electrode and the number in each frame is the time in ms. S_1_-S_2_ interval = 200 ms, S_2_ stimulus = 458 mA/cm. These frames are taken from the S8 Movie (see supplementary files).

**Fig 14:**
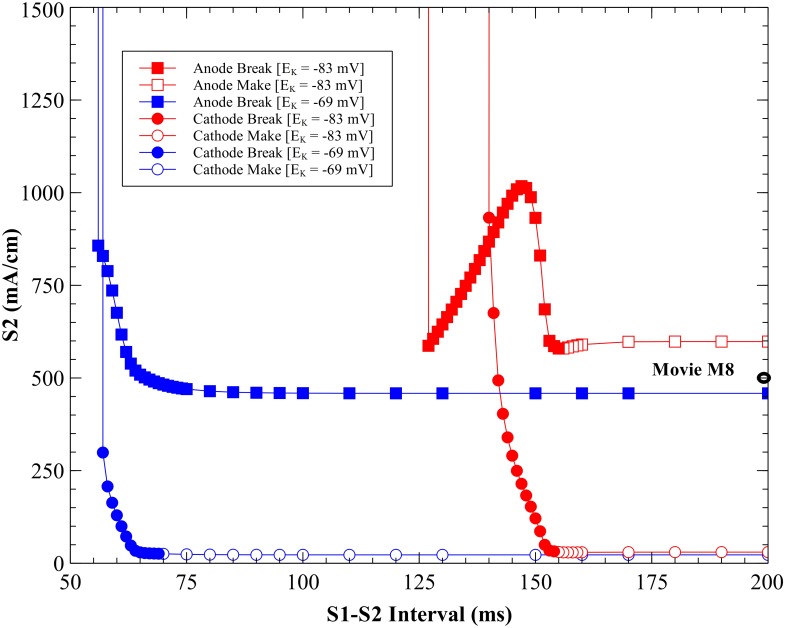
Comparison of SI curves for different *E_K_* values. Normal (*E_K_* = -83 mV red) and elevated (*E_K_* = -69 mV blue) extracellular potassium concentration. CM = cathode make (open circles), CB = cathode break (filled circles), AM = anode make (open squares), and AB = anode break (filled squares).

**Fig 15:**
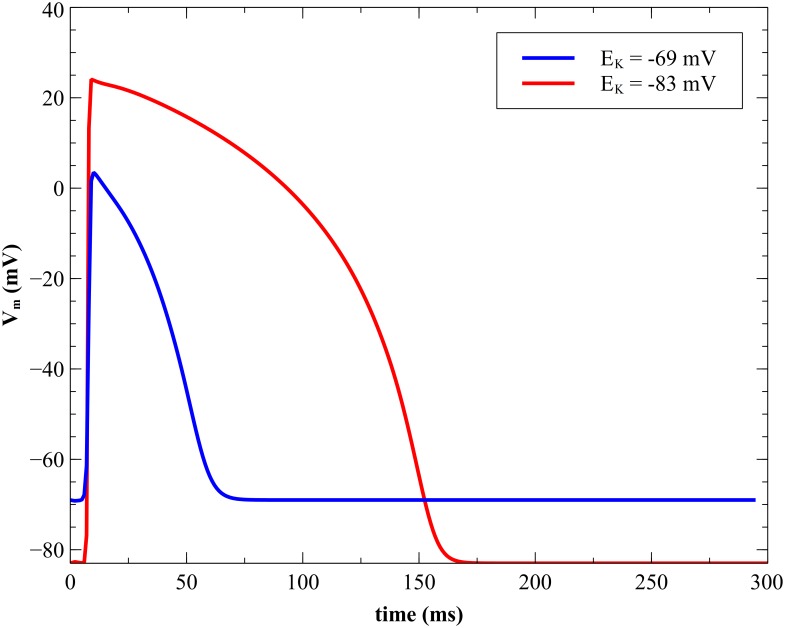
Transmembrane potential for normal and elevated potassium. *V_m_* vs time at *x* = 0.35 cm and *y* = 0.1 cm for different *E_K_* values (-83 mV normal red, and -69 mV elevated potassium blue).

At longer time intervals the primary mechanism of anodal excitation is make, whereas for shorter time intervals the primary mechanism is break [19]. These effects are evident in our calculations with the parsimonious ionic model (anode make in Fig 4 and anode break in Fig 5). When we increase the reversal potential (*E*_*K*_) to -69 mV, which represents an elevation in extracellular potassium ion concentration, at long time intervals the excitation mechanism is a combination of make and break (Fig 13 and S8 Movie, 200 ms, 500 mA/cm). Excitation with a weaker stimulus just above threshold (200 ms, 458 mA/cm) is purely break excitation, so we will categorize this more complex behavior as break. The change from make to break excitation at long intervals is consistent with the experimental work on elevated extracellular potassium ion concentration by Sidorov et al. [20], who showed that in diastole the anodal excitation changes from make to break. Numerical simulations carried out by Roth and Patel [21] using the Luo-Rudy dynamic model [8] suggest that potassium ion concentration influences anodal stimulation of cardiac tissue. Fig 14 shows the anodal and cathodal SI curves for *E*_*K*_ = -83 and -69 mV. The action potential duration shortens with an increase in *E*_*K*_ (Fig 15), causing the SI curves to shift to the left. Although the cathodal SI curve retains its shape the anodal SI curve for elevated potassium does not contain a dip. Therefore, elevated *E*_*K*_ suppresses the dip in the anodal SI curve, consistent with previous studies [20, 21]. It also suppresses the supernormal period.

## Discussion

Our simulations using the parsimonious ionic model for SI curves are consistent with results from previous studies [2,6,7,20,21]. The presence of the dip using a parsimonious model supports our hypothesis that the shape of the strength-interval curve is strongly influenced by the voltage dependent membrane recovery and bidomain properties of the tissue. By changing the parameter *k*_*r*_ in the parsimonious ionic model we were able to obtain SI curves with shorter APD yet which have a transition of anode break excitation to anode make excitation (for *k*_*r*_ = 21.28, 40 mV) as shown in movies in the supplementary files. The presence of the dip only at non-infinite values of *k*_*r*_ suggests that a nonlinear rectifying repolarization current is necessary for anode break excitation and the dip to occur in the anodal SI curve. Moreover, the parsimonious ionic model predicts the effect of elevated extracellular potassium concentration. Roth and Patel [21] found that the disappearance of the dip was caused by post-repolarization refractoriness, so that when the tissue recovered from refractoriness there was no S_1_ depolarization to assist with anode break excitation.

Why did we choose to use a parsimonious model rather than a more detailed model for the ionic current? Gray and Pathmanathan [13] make a compelling case for the utility of minimal models that contain few parameters. Most more detailed ionic models are derived from voltage clamp data obtained under non-physiological conditions. Complex models suffer from over-parameterization, non-uniqueness, and have limited validity beyond the data used in their derivation. The parsimonious model is mathematically identifiable [12] and exhibits important emergent phenomena such as alternans and spiral wave breakup [13]. This model is attractive for us because we wish to know if the shape of the strength-interval curve can be predicted using even a simple model of cardiac electrical behavior. Our conclusion is that the parsimonious model can predict the shape of the SI curve, and that more complicated and exotic ionic currents are not necessary to explain this behavior.

The parsimonious ionic model has its limitations. APD depends upon the degree of linearity of *I*_*K*_. The ideal model is such that we should be able to vary the degree of linearity without a change in APD, so that we can isolate the effect of nonlinearity. There is a discrepancy between the quantitative value of the threshold currents for SI curves compared to previous studies. The currents are relatively large for the parsimonious ionic model. There can be numerous reasons for this discrepancy [22].

In conclusion, a simple ion current model that includes only an excitable sodium current and a nonlinear repolarization current is sufficient to predict the dip in the anodal SI curve. The parsimonious ionic model with the bidomain tissue model can predict shapes of the anodal and cathodal SI curves and break and make excitations for both anodal and cathodal stimuli. Nonlinearity of the repolarization current and electrotonic interaction of depolarization and hyperpolarization are both necessary for a dip in the anodal strength-interval curve. Other researchers have postulated that the dip may be caused by the “funny current” that activates upon hyperpolarization *but that has a depolarized reversal potential* so it supplies inward current near rest potential [10,11], or by calcium dynamics [9]. Although we cannot rule out a role for such currents with our analysis, this parsimonious model indicates that such factors are not necessary to reproduce the observed strength-interval curves.

## Methods

The two dimensional bidomain model [23] is used to represent the cardiac tissue. The active membrane current is defined by the parsimonious ionic model [12, 13], which consists of two currents: a sodium current *I*_*Na*_ that activates rapidly upon depolarization, and a time-independent inward-rectifying repolarization current *I*_*K*_

$$I_{Na}=g_{Na}^{max}\;m^{3}h\left(V-\;E_{Na}\right)$$ (1)

$$I_{K}=g_{K}\left(V-\;E_{K}\right)\text{exp}\left[\frac{-\left(V-E_{K}\right)}{k_{r}}\right]$$ (2)

where

$$\frac{dm}{dt}\;=\;\frac{m_{\infty }\left(V\right)-m}{\tau_{m\left(V\right)}}\;,$$ (3)

$$\frac{dh}{dt}\;=\;\frac{h_{\infty }\left(V\right)-h}{\tau_{h}(V)}\;,$$ (4)

with

$$m_{\infty }(V)\;=\;\frac{1}{1+\text{exp}\left[\frac{-(V-E_{m})}{k_{m}}\right]}$$ (5)

$$h_{\infty }(V)\;=\;\frac{1}{1+\text{exp}\left[\frac{+(V-E_{h})}{k_{h}}\right]}$$ (6)

$$\tau_{m}\left(V\right)=\;\tau_{mo}$$ (7)

$$\tau_{h}\left(V\right)=\;\frac{2\tau_{ho}exp\left[\frac{\delta_{h}\left(V-E_{h}\right)}{k_{h}}\right]}{1+\text{exp}\left[\frac{\left(V-E_{h}\right)}{k_{h}}\right]}\;.$$ (8)

The bidomain model is,

$$g_{ex}\;\frac{\partial^{2}V_{e}}{\partial x^{2}}+\;g_{ey}\;\frac{\partial^{2}V_{e}}{\partial y^{2}}=\;-\beta \left[C_{m}\frac{\partial V_{m}}{\partial t}+\;I_{Na}+\;I_{K}\right]$$ (9)

$$\left(g_{ix}+g_{ex}\right)\frac{\partial^{2}V_{e}}{\partial x^{2}}+\left(g_{iy}+g_{ey}\right)\frac{\partial^{2}V_{e}}{\partial y^{2}}=-\left(g_{ix}\;\frac{\partial^{2}V_{m}}{\partial x^{2}}+\;g_{iy}\;\frac{\partial^{2}V_{m}}{\partial y^{2}}\right)\;\;,$$ (10)

where *V*_*m*_ and *V*_*e*_ are the transmembrane and extracellular potentials, *C*_*m*_ is the membrane capacitance per unit area, and *β* is the surface-to-volume ratio. Values for the intracellular and extracellular conductivities in the directions parallel and perpendicular to the fibers, *g*_*ix*_, *g*_*iy*_, *g*_*ex*_, *g*_*ey*_ (Table 1), are chosen such that the tissue has unequal anisotropy ratios [16]. The stimulus current is applied at the center of the tissue using a rectangular unipolar electrode having a length (*x*-direction) and width (*y*-direction) of 0.1 cm. The stimulus is applied to a tissue of 1.0 cm by 1.0 cm by applying boundary conditions to the electrode tissue surface: we consider that the total current applied to the electrode is transferred to the extracellular space as the stimulating current, the normal component of the intracellular current density is zero, and the extracellular potential is kept constant over the electrode surface. All stimuli have a duration of 2 ms. Before stimulation the resting potential is equal to *E*_*K*_. In each simulation, the initial stimulus (S_1_) is cathodal with twice the rheobase stimulus strength. Rheobase depends on the model parameters, so the S_1_ strength in mA/cm varies between simulations.

**Table 1:** Parameters in the model.

$g_{Na}^{max}=11\;{\text{mS cm}}^{-2}$	*k*_*h*_ = 4.4 mV
*E*_*Na*_ = 65 mV	*τ*_*ho*_ = 6.80738 ms
*E*_*K*_ = −83 mV	*δ*_*h*_ = 0.799163
*g*_*K*_ = 0.3 mS cm^−2^	*β* = 2000 cm^−1^
*k*_*r*_ = 21.28 mV	*C*_*m*_ = 1 μF cm^−2^
*E*_*m*_ = −41 mV	*g*_*ix*_ = 2 mS cm^−1^
*k*_*m*_ = 4 mV	*g*_*iy*_ = 0.2 mS cm^−1^
*τ*_*m*_ = 0.12 ms	*g*_*ex*_ = 8 mS cm^−1^
*E*_*h*_ = −74.7 mV	*g*_*ey*_ = 2 mS cm^−1^

All movies were generated using 1 ms per frame. Only one quadrant of the tissue is shown. The transmembrane potential in the other quadrants can be found from the even symmetry. Movies were generated using an S_2_ strength about 10% above the threshold strength for that interval.

Space-clamped simulations were performed using Eq 9 with the left-hand-side set to zero, and the stimulus was applied through an additional membrane current added to *I*_*K*_ and *I*_*Na*_.

When equal anisotropy ratios were used, the conductivities were chosen to be, *g*_*ix*_ = *g*_*ex*_ = 2 mS cm^-1^, and *g*_*iy*_ = *g*_*ey*_ = 0.32 mS cm^-1^.

The bidomain equations are solved numerically with the finite difference method. Using an initial value of *V*_*e*_(*t*), we solve Eq 9 for *V*_*m*_(*t+Δt*). Then we solve Eq 10 for *V*_*e*_(*t+Δt*) using the value of *V*_*m*_(*t+Δt*) in the source term, and the successive overrelaxation (SOR) method [4,6]. In order to accelerate convergence, an overrelaxation parameter of *w* = 1.8 was used [24]. The iterative loop terminates when changes in *V*_*e*_ between subsequent time steps is less than 1 μV. All programs are written in Fortran 90 and compiled using the gfortran compiler (www.gcc.gnu.org). The space step is 0.005 cm parallel to the fiber direction and 0.002 cm perpendicular to the fiber direction. The time step is 1 μs. The number of nodes in *x*-direction is 200 and the number of nodes in *y*-direction is 500. Because the tissue is stimulated from the center and propagation is symmetrical, we consider only one quadrant of the tissue.

**Fig 16:**
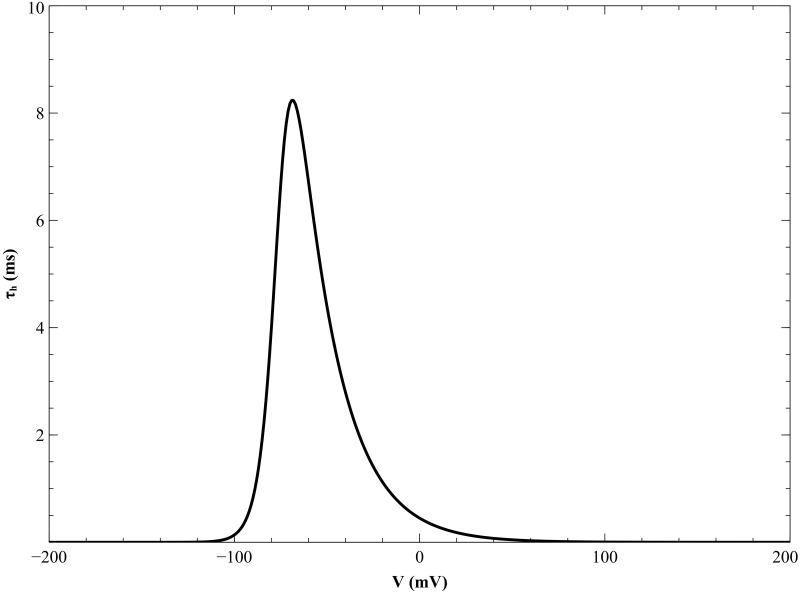
Variation of *τ_h_* with transmembrane potential.

During simulations of anodal excitation, we encountered a computational problem arising from the strong hyperpolarization next to the electrode. The time constant *τ*_*h*_ became too small (even smaller than the time step), making the calculations unstable (Fig 16). In order to correct this, we modified the parsimonious ionic model by requiring that the minimum value of *τ*_*h*_ be the time step (1 μs). This modification made the simulations stable.

## Supporting information

S1 Movie Cathode make excitation. S_1_-S_2_ interval = 155 ms, S_2_ stimulus strength = 33 mA/cm, S_2_ stimulus polarity = cathodal.
(MP4)

S2 Movie Cathode break excitation. S_1_-S_2_ interval = 143 ms, S_2_ stimulus strength = 435 mA/cm, S_2_ stimulus polarity = cathodal.
(MP4)

S3 Movie Anode make excitation. S_1_-S_2_ interval = 158 ms, S_2_ stimulus strength = 640 mA/cm, S_2_ stimulus polarity = anodal.
(MP4)

S4 Movie Anode break excitation. S_1_-S_2_ interval = 130 ms, S_2_ stimulus strength = 710 mA/cm, S_2_ stimulus polarity = anodal.
(MP4)

S5 Movie Failed anode break excitation. S_1_-S_2_ interval = 125 ms, S_2_ stimulus strength = 600 mA/cm, S_2_ stimulus polarity = anodal.
(MP4)

S6 Movie Anode break excitation. S_1_-S_2_ interval = 130 ms, S_2_ stimulus strength = 750 mA/cm, S_2_ stimulus polarity = anodal. *k*_*r*_ = 21.28 mV and g_K_ = 0.3 mS cm^-2^.
(MP4)

S7 Movie Failed anode break excitation. S_1_-S_2_ interval = 130 ms, S_2_ stimulus strength = 750 mA/cm, S_2_ stimulus polarity = anodal. *k*_*r*_ = ∞ and g_K_ = 0.016 mS cm^-2^.
(MP4)

S8 Movie Anode make/break excitation for *E*_*K*_ = -69 mV. S_1_-S_2_ interval = 200 ms, S_2_ stimulus strength = 500 mA/cm, S_2_ stimulus polarity = anodal.
(MP4)
